# The Usefulness of Lung Ultrasound for the Aetiological Diagnosis of Community-Acquired Pneumonia in Children

**DOI:** 10.1038/s41598-019-54499-y

**Published:** 2019-11-29

**Authors:** Vojko Berce, Maja Tomazin, Mario Gorenjak, Tadej Berce, Barbara Lovrenčič

**Affiliations:** 1 University Medical Centre Maribor, Division of Pediatrics, Ljubljanska ulica 5, 2000 Maribor, Slovenia; 2 Faculty of Medicine, University of Maribor, Taborska ulica 8, 2000 Maribor, Slovenia; 3 Faculty of Medicine, University of Ljubljana, Vrazov trg 2, 1000 Ljubljana, Slovenia

**Keywords:** Diagnostic markers, Infectious diseases, Paediatric research

## Abstract

The aetiology of community-acquired pneumonia (CAP) is not easy to establish. As lung ultrasound (LUS) has already proved to be an excellent diagnostic tool for CAP, we analysed its usefulness for discriminating between the aetiologically different types of CAP in children. We included 147 children hospitalized because of CAP. LUS was performed in all patients at admission, and follow-up LUS was performed in most patients. LUS-detected consolidations in viral CAP were significantly smaller, with a median diameter of 15 mm, compared to 20 mm in atypical bacterial CAP (p = 0.05) and 30 mm in bacterial CAP (p < 0.001). Multiple consolidations were detected in 65.4% of patients with viral CAP and in 17.3% of patients with bacterial CAP (p < 0.001). Bilateral consolidations were also more common in viral CAP than in bacterial CAP (51.9% vs. 8.0%, p < 0.001). At follow-up, a regression of consolidations was observed in 96.6% of patients with bacterial CAP and in 33.3% of patients with viral CAP (p < 0.001). We found LUS to be especially suitable for differentiating bacterial CAP from CAP due to other aetiologies. However, LUS must be interpreted in light of clinical and laboratory findings.

## Introduction

Childhood community-acquired pneumonia (CAP) is a common infection of the lower respiratory tract and the single most important cause of mortality in preschool children in the developing world^1^. In the developed world, CAP is imposing a significant burden of morbidity, with an estimated annual incidence of 14.5 per 10,000 children from 0 to 16 years old^2^. The diagnosis of CAP in children can be challenging as there is no pathognomonic sign or symptom^3^.

Respiratory viruses are the most common cause of CAP in preschool children, followed by bacteria, especially *Streptococcus pneumoniae*. The atypical bacteria *Mycoplasma pneumoniae* and *Chlamydophila pneumoniae* are common causes of pneumonia in children older than 5 years^4^. The identification of the causal agent is pivotal, especially in children who require hospital admission, as it guides the choice of appropriate treatment. However, the microbial diagnosis of CAP in children is not easy to establish without invasive procedures, which are only rarely performed in this age group^2,5^. Pneumonia can be a life-threatening disease if left untreated^6^. Initially, antibiotic therapy is empirical and influenced by epidemiological, clinical and radiographic findings. Slovenian guidelines recommend a penicillin-based antibiotic as a first-line therapy for non-complicated bacterial CAP in the paediatric population. Macrolide antibiotics should be used for the presumed atypical bacterial CAP^7^. Children with non-complicated viral CAP need only supportive treatment^6^.

Clinical features of bacterial pneumonia, atypical bacterial pneumonia or viral pneumonia frequently overlap and cannot be used reliably to distinguish between the various aetiologies^8^. The same applies to blood tests such as the complete blood count (CBC) with differential and acute phase reactants. Normal white blood cell (WBC) count and low C-reactive protein (CRP) do not exclude bacterial CAP^6^. On the other hand, a low serum procalcitonin (PCT; <0.25 ng/ml) was recently found to have a 96% negative predictive value (95% confidence interval [CI], 93–99), 85% sensitivity (95% CI, 76–95), and 45% specificity (95% CI, 40–50) in identifying children without typical bacterial CAP^9^.

Chest X-ray (CXR) is not necessary to confirm the diagnosis of CAP in patients with milder disease, who are treated as outpatients and are also associated with a small, albeit not completely negligible, risk of radiation exposure^10^. Although CXR is not considered a “gold standard”, it has a high negative predictive value for CAP in children^11^. However, CXR cannot reliably establish the microbial diagnosis of CAP^2^, and the interpretation of radiographic images varies significantly among the observers^12^. Nevertheless, there are some radiographic characteristics that are more often associated with the specific microbial aetiology of CAP. Alveolar infiltrate in the form of lobar, segmental or round consolidation is relatively specific for bacterial pneumonia but lacks sensitivity^13^. Viral pneumonia often presents with bilateral interstitial infiltrates on CXR^14^. A similar form of infiltrates can be observed in atypical bacterial CAP^15^. However, infection with *M*. *pneumoniae* often radiologically mimics classic bacterial CAP, presenting with alveolar infiltrate or even small pleural effusion^6,16^.

Lung ultrasound (LUS) seems to be a sufficiently accurate technique for diagnosing pneumonia in the paediatric population with high sensitivity and specificity and may represent an alternative diagnostic tool to CXR^17–20^. The advantages of LUS are as follows: no ionizing radiation, lower cost, the possibility of follow-up examination, the ability to monitor the effect of therapy, and better patient cooperation^21,22^. Furthermore, this diagnostic technique is accessible, portable, fast, easy to learn, and can be used immediately as a point-of-care method. LUS has good diagnostic accuracy even when performed by non-experts^18,20^. By using LUS, it is possible to observe many pathological lung patterns associated with pneumonia, such as consolidation, pleural effusion, and interstitial syndrome. Consolidation, as seen on LUS, is hypoechoic or isoechoic, has a tissue-like structure and represents a loss of lung aeration. Branching, hyperechoic and dynamic air bronchograms detected within the area of consolidation, is the hallmark of pneumonia^17,23^.

Anechoic fluid bronchograms are also characteristic of pneumonic consolidation but are only very seldom encountered without the air bronchograms in children with CAP^24^. Static air bronchograms are more a characteristic of lung collapse but can also be present in pneumonic consolidation. In such cases, it is difficult to distinguish between pneumonia and lung collapse^25^.

Studies using LUS for the identification of bacterial superinfection in patients with viral lower respiratory tract infection (LRTI) have already been performed and considered small subpleural consolidations and/or an increased number of B-lines (interstitial syndrome) as characteristics of viral pneumonia. A similar LUS pattern can also be encountered in viral bronchiolitis^26,27^. Urbankowska *et al*. found a positive correlation between the size of LUS-detected consolidations and neutrophil count, which implies the association of larger consolidations with bacterial CAP. They also found LUS to be very useful for the follow-up of CAP in children and observed a complete regression of consolidations in 44% of patients after 5–7 days of treatment^19^. However, to the best of our knowledge, our study is the first to investigate the usefulness of LUS in the aetiological diagnosis of all types of CAP simultaneously.

Therefore, the aim of the present study was to find an association between LUS characteristics and the aetiological diagnosis of CAP in the paediatric population. By analogy to the CXR characteristics of different types of CAP, we hypothesize that the aetiologically different types of CAP (bacterial, viral, atypical bacterial) differ in their LUS characteristics. More specifically, the consolidations in bacterial CAP are more likely to be solitary, larger and unilateral than those in the viral and atypical bacterial CAPs, where we expect to find multiple consolidations that are smaller and more often bilateral.

## Participants and Methods

### Participants

**Figure 1:**
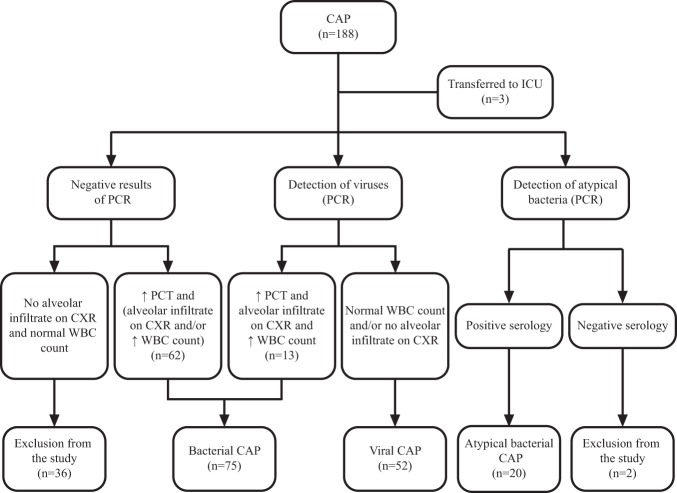
Stratification of patients with community-acquired pneumonia according to the aetiology. CAP: community-acquired pneumonia; PCR: polymerase chain reaction-based assay from nasopharyngeal swab; ↑ PCT: increased serum procalcitonin concentration (>0.25 ng/ml); ↑ WBC: increased white blood cell count (>15 × 10^9^/L); CXR: chest X-ray. Thirty-six patients were excluded from the study because we could not determine the aetiology. Two patients were excluded from the study because of lack of the serologic confirmation of *Mycoplasma pneumoniae* infection and three patients because of the transfer to the intensive care unit. Thirteen patients in whom viruses were detected in the nasopharynx were classified as having bacterial CAP (co-infection or superinfection).

We performed a prospective study and included 147 children with CAP who were hospitalized in our Department of Pediatrics from October 1, 2014 to September 30, 2018. The age of the patients ranged from 1 month to 16 years. Initially we enrolled all (188) children who were hospitalized in the aforementioned period for CAP and had pneumonic consolidation(s) detected with LUS. LUS was performed in all patients with at least two of the following signs and symptoms: fever (>38 °C), cough, dyspnoea, abnormal auscultatory findings, chest or abdominal pain. LUS was also performed in children with a fever without a localizing signs and leucocytosis (>15 × 10^9^/L). All the studied children were previously healthy and were not born premature. We excluded patients with immune deficiency, neurological impairment, chronic lung (except asthma) or heart disease or any other chronic condition that can predispose individuals to pneumonia. In addition, we excluded patients with severe CAP who required management in the paediatric intensive care unit (ICU). Some patients were excluded only after the completion of treatment, when alternative diagnoses were established or when we were unable to determine the aetiology of CAP. Final number of included patients for subsequent analysis was 147 (Fig. 1). We did not exclude patients who were already treated with antibiotics before admission. There were 12 (9.3%) such patients, 7 (8.2%) of whom were in the bacterial CAP group. The diagnosis of CAP was confirmed according to the British Thoracic Society (BTS) criteria at discharge from the hospital by two senior paediatric pulmonologists^2^.

### Ethical approval and informed consent

The study was approved by the Ethics Committee of University Medical Centre, Maribor, Slovenia. All methods were performed in accordance with the relevant guidelines and regulations. Legal guardians of all participants signed an informed consent form according to the World Medical Association Declaration of Helsinki, revised in 2000, Edinburgh. There is no identifying information or image in the article.

### Diagnostic investigations

Venous blood was collected from all participants to obtain complete and differential blood count and CRP levels. PCT levels were determined, and a blood culture was performed in patients with suspected bacterial CAP. We collected nasopharyngeal swabs for the detection of the most common respiratory viruses and three atypical bacteria using polymerase chain reaction (PCR)-based assays from all patients. We tested for the presence of respiratory syncytial virus (RSV), human rhinovirus, human bocavirus (HBoV), influenza A, influenza B, parainfluenza viruses (serotypes 1, 2, 3 and 4), adenovirus, human metapneumovirus (HMPV), enterovirus, coronavirus, *M*. *pneumoniae*, *Bordetella pertussis* and *C*. *pneumoniae*. We confirmed acute infection with *Mycoplasma pneumoniae* by detecting the specific M class antibody (IgM) in the convalescent phase using enzyme-linked immunosorbent assays (ELISAs)^28^. All microbiological assays were performed by the National Laboratory of Health, Environment and Food, Maribor, Slovenia.

All 147 patients underwent LUS on the day of admission, followed by CXR in 120 patients. LUS was repeated after 48–72 hours in 111 (75.5%) patients who were still hospitalized at that time. CXR was performed in all patients with uncertain aetiology (e.g. detected viruses and leucocytosis) and at the discretion of the physician. A standard posteroanterior (PA) view was used in the CXR, and the image was evaluated by a paediatric radiologist.

### Lung ultrasound

LUS was performed with the portable ultrasound machine Sonosite (SonoSite, Inc. Bothell, WA, USA) by a paediatric pulmonologist who was unaware of the clinical and laboratory data and of the CXR results. A linear probe (13–6 MHz) was used in preschool children. In older children, we used a curved probe (8–5 MHz). Infants and toddlers were examined in the upright position in the arms of one of their parents, and older patients were seated. LUS was performed according to the technique described by Copetti and Cattarossi^23^. Only the B-mode was used, and Doppler ultrasound was performed for the evaluation of the blood perfusion of the affected lung tissue. Cine loops were obtained and later discussed with another paediatric pulmonologist. Pleural effusion and the increased number of B-lines (≥3 per intercostal space) were also recorded. However, only the presence of consolidation was considered a diagnostic criterion for pneumonia in our study. Pneumonic consolidation was defined as the presence of hypoechoic or isoechoic (echogenicity similar to liver) areas with dynamic air bronchogram and/or shred sign to distinguish between the pneumonic consolidation and lung collapse^29,30^.

When more than one discrete lung consolidation was detected with LUS simultaneously, we considered the patient to have multiple consolidations for the purpose of statistical analysis, except for the calculation of correlation, where the actual number of consolidations was taken into account. We considered the presence of bilateral consolidations when consolidations were detected with LUS on both lungs simultaneously. The dimensions of each consolidation were measured in the longitudinal, transverse and sagittal axes, and the largest diameter was recorded. At follow-up, the LUS regression/progression of consolidations in size and number was evaluated, and patients were stratified at the discretion of the physician who performed the LUS into four groups: progression, no regression, regression, and complete resolution. In addition, consolidation(s) were measured again as described above.

### Stratification of patients

Patients were stratified into the three different groups according to the presumed microbial aetiology (Fig. 1). Patients with detected viral infection were stratified into the viral CAP group only after the exclusion of bacterial superinfection. Bacterial CAP was excluded in all patients with normal serum PCT (<0.25 ng/ml)^9^. Bacterial CAP (co-infection or superinfection) was considered in patients with leucocytosis (>15 × 10^9^/L) and alveolar infiltrate(s) on CXR, even when viruses were detected in the nasopharyngeal swab. When no viruses or atypical bacteria were detected, leucocytosis or alveolar infiltrate alone was enough for the stratification into the bacterial CAP group^13,31^. Bacterial CAP was also considered in all patients with positive blood culture. When no aetiology could be established (negative nasopharyngeal swab, normal WBC count, absence of alveolar infiltrates on CXR), the patient was excluded from the study.

### Statistical analysis

Statistical analysis was performed with IBM SPSS 24.0 software (IBM Inc., Chicago, IL, USA). The Mann-Whitney U-test was performed to compare quantitative variables between the different CAP groups and after a Kolmogorov-Smirnov test of normality. The association of the aetiology of CAP with qualitative clinical, CXR and LUS characteristics was analysed using Fischer’s exact or chi-squared test. Risk, positive predictive value (PPV) and negative predictive value (NPV) were calculated for the bacterial CAP. A receiver-operating characteristic (ROC) curve analysis was applied to assess the optimal consolidation size cut-off value for discriminating between the different types of CAP. Correlations between quantitative variables were analysed with Spearman’s rank correlation coefficient, and the agreement between the CXR and LUS regarding the presence of bilateral infiltrates was analysed with Cohen’s kappa coefficient. We considered the strength of the correlation to be very weak (0.0–0.19), weak (0.20–0.39), moderate (0.40–0.59), strong (0.60–0.79) or very strong (0.80–1.0). A comparison of ultrasound characteristics, adjusted for age, sex, and clinical and laboratory characteristics, was performed with logistic regression. The α level for all tests was set to 0.05, and P values are presented for two-tailed tests.

## Results

Raw data, including the demographic and clinical characteristics as well as the laboratory, CXR, LUS and microbiological results of patients, are presented in the Supplement (Supplementary dataset).

### Demographic, clinical and laboratory characteristics

We included 73 (49.7%) females and 74 (50.3%) males.

Pneumonia was caused by bacteria, atypical bacteria and viruses in 75 (51.0%), 20 (13.6%) and 52 (35.4%) subjects, respectively.

The demographic, clinical and laboratory characteristics of participants according to the aetiology of CAP are presented in Table 1. Blood culture was positive in only 4 patients (5.3% of all subjects with bacterial CAP), and *Streptococcus pneumoniae* was isolated in all cases.

**Table 1:** Clinical and laboratory characteristics of patients with different types of pneumonia.

Characteristic [n (%)]*	Bacterial CAP (n = 75)	Atypical CAP (n = 20)	Viral CAP (n = 52)	p value**	Odds ratio (95% confidence interval)***	Positive predictive value (%)****	Negative predictive value (%)****
Crackles on auscultation	24 (32.0)	18 (90.0)	41 (78.8)	BA < 0.001 AV 0.330 BV < 0.001	BA 0.05 (0.01–0.24) AV 2.46 (0.49–12.02) BV 0.13 (0.06–0.29)	28.9	20.3
Wheezes on auscultation	6 (8.0)	3 (15.0)	22 (42.3)	BA 0.392 AV 0.051 BV < 0.001	BA 0.49 (0.11–2.17) AV 0.24 (0.06–0.92) BV 0.12 (0.04–0.32)	19.4	40.5
Chest/abdominal pain	37 (55.2)	6 (30.0)	5 (12.5)	BA 0.073 AV 0.155 BV < 0.001	BA 2.88 (0.99–8.40) AV 3.00 (0.79–11.45) BV 8.63 (3.01–24.76)	77.1	62.0
Diminished breath sounds	24 (32.0)	4 (20.0)	7 (13.5)	BA 0.410 AV 0.485 BV 0.021	BA 1.88 (0.57–6.24) AV 1.61 (0.42–6.23) BV 3.03 (1.19–7.69)	68.6	54.5
Bronchial breathing	15 (20.0)	1 (5.0)	0 (0.0)	BA 0.179 AV 0.278 BV < 0.001	BA 4.75 (0.59–38.36) AV 3.74 (2.54–5.39) BV 1.87 (1.57–2.22)	93.8	54.2
Need of additional oxygen	5 (6.7)	5 (25.0)	27 (51.9)	BA 0.032 AV 0.063 BV < 0.001	BA 0.21 (0.06–0.83) AV 0.31 (0.10–0.97) BV 0.07 (0.02–0.19)	13.5	36.4
**Characteristic [median (IQR)]**							
Age (months)	42 (38)	85 (76)	26 (27)	BA 0.006 AV < 0.001 BV < 0.001			
WBC count (x 10^9^/L)	22.1 (11.7)	10.8 (4.6)	13.7 (10.6)	BA < 0.001 AV 0.98 BV < 0.001			
CRP (mg/dL)	149 (103)	17.5 (37)	44 (79)	BA < 0.001 AV 0.053 BV < 0.001			

*number of subjects with a particular characteristic (percentage in parentheses).

**p value refers to the comparison between bacterial and atypical bacterial CAP (BA), between atypical bacterial and viral CAP (AV) and between bacterial and viral CAP (BV); chi-squared or Fischer’s exact text was used for the comparison of qualitative variables and Mann-Whitney U-test was used for the comparison of quantitative variables.

***Odds ratio is calculated for bacterial pneumonia (BA and BV) or atypical bacterial pneumonia (AV).

****Positive and negative predictive value is calculated for bacterial pneumonia.

*****Chest and/or abdominal pain was recorded only in patients aged at least 15 months (67 patients with bacterial CAP, 20 with atypical bacterial CAP and 40 with viral CAP).

CAP: community-acquired pneumonia; IQR: interquartile range; WBC: white blood cell; CRP: C-reactive protein.

### Chest X-ray

CXR was performed in 120 (81.6%) patients with CAP; pneumonic infiltrates were detected in 92 (76.7%) of them. Infiltrates were detected in 50 (79.4% of those who underwent CXR) patients with bacterial CAP, in 12 (80.0%) patients with atypical bacterial CAP and in 30 (71.4%) patients with viral CAP. The radiological characteristics of patients who had CAP detected with CRX (in addition to LUS) are presented in Table 2.

**Table 2:** Chest X-ray characteristics of patients with different types of pneumonia.

Characteristic [n (%)]*	Bacterial CAP n = 50	Atypical CAP n = 12	Viral CAP n = 30	p value**	Odds ratio (95% confidence interval)***	Positive predictive value (%)****	Negative predictive value (%)****
Unilateral infiltrate(s)	46 (92.0)	9 (75.0)	20 (66.7)	BA 0.125 AV 0.722 BV 0.006	BA 3.83 (0.73–20.13) AV 1.50 (0.33–6.80) BV 5.75 (1.61–20.53)	61.3	76.5
Alveolar infiltrate(s)	42 (84.0)	4 (33.3)	10 (33.3)	BA 0.001 AV 1.000 BV < 0.001	BA 10.50 (2.54–43.36) AV 1.00 (0.24–4.14) BV 10.50 (3.60–30.65)	75.0	77.8
Pleural effusion	2 (3.2)	2 (13.3)	2 (4.8)	BA 0.165 AV 0.281 BV 1.000	BA 0.21 (0.03–1.65) AV 3.08 (0.39–24.08) BV 0.66 (0.09–4.85)	33.3	46.5

*Number of subjects with a particular characteristic (percentage in parentheses).

**p value refers to the comparison between bacterial and atypical bacterial CAP (BA), between atypical bacterial and viral CAP (AV) and between bacterial and viral CAP (BV); chi-squared or Fisher’s exact test was used.

***Odds ratio is calculated for bacterial pneumonia (BA and BV) or atypical bacterial pneumonia (AV).

****Positive and negative predictive value – calculated for bacterial pneumonia.

CAP: community-acquired pneumonia.

### Lung ultrasound

LUS was performed at admission in all 147 patients; multiple consolidations were detected in 60 (40.8%) and bilateral consolidations in 42 (28.6%) of them. A comparison of LUS characteristics in different types of CAP is presented in Table 3.

**Table 3:** Comparison of ultrasound characteristics of different types of pneumonia.

Characteristic [n (%)]*	Bacterial CAP	Atypical CAP	Viral CAP	p value**	Odds ratio (95% confidence interval)***	Positive predictive value (%)****	Negative predictive value (%)****
Unilateral consolidation	69 (92.0)	11 (55.0)	25 (48.1)	BA < 0.001 AV 0.793 BV < 0.001	BA 9.41 (2.80–31.66) AV 1.32 (0.47–3.72) BV 12.42 (4.59–33.62)	65.7	85.7
Solitary consolidation	62 (82.7)	7 (35.0)	18 (34.6)	BA < 0.001 AV 1.000 BV < 0.001	BA 8.86 (2.96–26.51) AV 1.02 (0.35–3.00) BV 9.01 (3.94–20.60)	71.3	78.3
Pleural effusion	14 (18.7)	3 (15.0)	2 (3.8)	BA 1.000 AV 0.127 BV 0.014	BA 1.30 (0.34–5.06) AV 4.41 (0.68–28.68) BV 5.74 (1.25–26.45)	73.7	52.3
**Characteristic [median (IQR)]**							
Largest consolidation diameter on admission (mm)	30 (20)	20 (23)	15 (14)	BA 0.12 AV 0.05 BV < 0.001			
Largest consolidation diameter at follow-up (mm)	14.5 (20)	8 (16)	12 (5)	BA 0.801 AV 0.356 BV 0.490			

*Number of subjects with a particular characteristic (percentage in parentheses).

**p value refers to the comparison between bacterial and atypical bacterial CAP (BA), between atypical bacterial and viral CAP (AV) and between bacterial and viral CAP (BV); chi-squared or Fischer’s exact text was used for the comparison of qualitative variables and Mann-Whitney U-test was used for the comparison of quantitative variables.

***Odds ratio is calculated for bacterial pneumonia (BA and BV) or atypical bacterial pneumonia (AV).

****Positive and negative predictive value is calculated for bacterial pneumonia.

CAP: community-acquired pneumonia.

**Figure 2:**
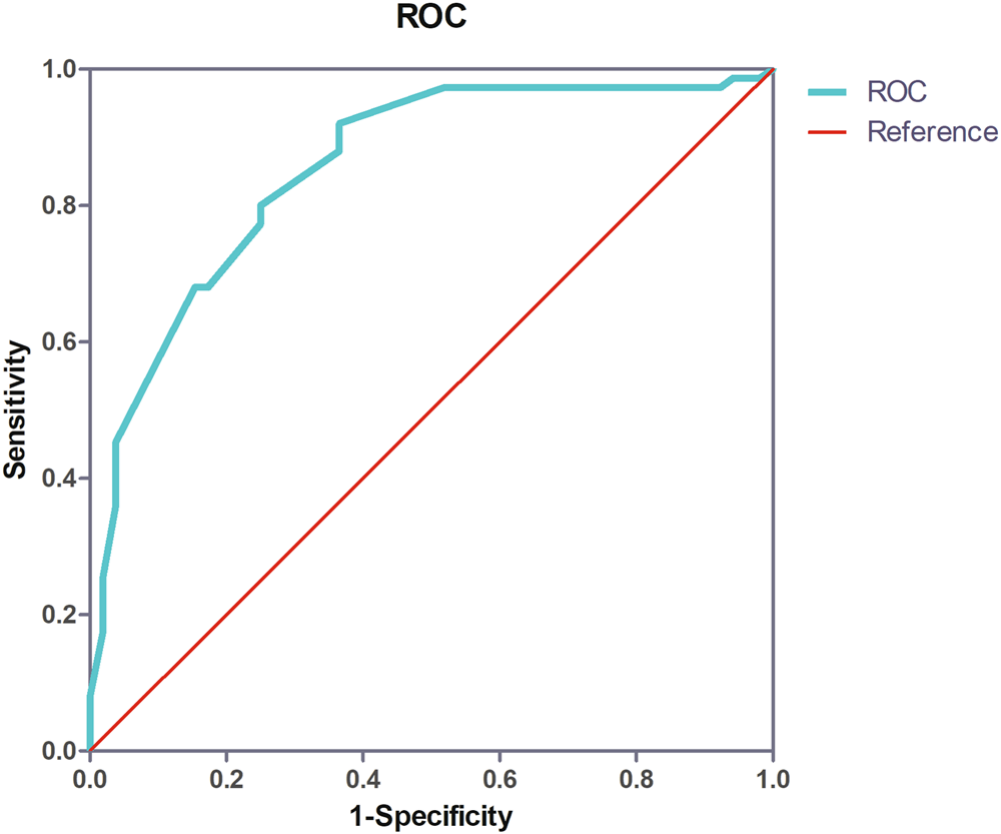
A receiver operating characteristics (ROC) curve analysis of the lung-ultrasound-detected consolidation size, discriminating between the bacterial and viral pneumonia. The optimal cut-off size was 21 mm, with a sensitivity of 80% and a specificity of 75% to diagnose bacterial CAP. The area under the ROC curve (AUC) was 0.85 (p < 0.001; 95% CI 0.79–0.92).

The ROC curve analysis (Fig. 2) found that the optimal cut-off for discriminating between the bacterial and viral CAP is a consolidation size of 21 mm, with a sensitivity of 80% and a specificity of 75% for diagnosing bacterial CAP. The area under the ROC curve (AUC) was 0.85 (95% CI 0.79–0.92, p < 0.001). Similarly, the optimal cut-off for discriminating between the bacterial and atypical bacterial CAP is a consolidation size of 21 mm, with a sensitivity of 80% and a specificity of 60% to diagnose bacterial CAP. The area under the ROC curve was 0.68 (95% CI 0.52–0.85, p = 0.012). Regarding the discrimination between atypical bacterial CAP and viral CAP, the AUC was 0.65 (95% CI 0.50–0.80, p = 0.051).

A weak positive correlation was found between the size of the (largest) consolidation and the WBC count (ρ = 0.28, p < 0.001).

Single consolidation, two consolidations, three consolidations and four (or more) consolidations were detected with LUS in 87 (59.2%), 21 (14.3%), 14 (9.5%) and 25 (17.0%) patients, respectively. A weak to moderate negative correlation was observed between the number of consolidations and the WBC count (ρ = −0.35, p < 0.001).

A significant agreement was found between the LUS and CXR regarding the presence of bilateral consolidations (κ = 0.45, p < 0.001).

Follow-up LUS was performed in 111 (75.5%) patients, of whom 58, 14 and 39 patients had bacterial, atypical bacterial and viral CAP, respectively. A regression of consolidations was observed in 56 (96.6%), 7 (50.0%) and 13 (33.3%) patients with bacterial, atypical bacterial and viral CAP, respectively. Of these, we found a complete resolution of consolidations in 16 (27.6%), 2 (14.3%) and 1 (2.6%) patient with bacterial, atypical bacterial and viral CAP, respectively. A regression of consolidations was significantly more common in bacterial CAP than in viral CAP (p < 0.001, OR = 56.00 with 95% CI 11.77–266.41) and in bacterial CAP than in atypical bacterial CAP (p < 0.001, OR = 28.00 with 95% CI 4.83–162.25). There was no significant difference in the regression of consolidations between atypical bacterial and viral CAP (p = 0.341, OR = 0.50 with 95% CI 0.15–1.73).

In addition, we analysed the association of LUS characteristics with the aetiology of CAP (viral vs. bacterial) using a regression model to adjust for age, sex, and laboratory characteristics. The regression model was statistically significant (p < 0.001), with 83.6% of the dependent variable variability explained. The accuracy of the model was 90.9%. The size of the largest consolidation in the model was still significant for bacterial CAP (p < 0.001; OR = 1.13 with 95% CI 1.04–1.23). The presence of bilateral consolidations also remained significant (p = 0.042; OR = 0.05 with 95% CI 0.01–0.86) in favour of a decreased probability for bacterial CAP. When all the clinical characteristics were included in the logistic regression analysis, the regression model remained highly significant (p < 0.001), and the accuracy of the model increased to 93.3%. The size of the consolidation remained significant for bacterial pneumonia (p = 0.041; OR = 1.09 with 95% CI 1.01–1.19).

## Discussion

In our prospective study, we have shown that LUS not only is a sensitive tool for detection but also can contribute to the aetiological diagnosis of CAP in children.

We found that patients with viral or atypical bacterial CAP were more likely to have multiple consolidations simultaneously detected with LUS than bacterial CAP, where solitary consolidations predominated. In addition, consolidations in patients with viral or atypical bacterial CAP were smaller and more likely bilateral. These findings are in concordance with the studies performed previously with CXR, where large alveolar infiltrates have shown a good positive predictive value for bacterial pneumonia^32^ and bilateral interstitial infiltrates were a characteristic of viral CAP^14^. Studies of the usefulness of CXR in the microbial diagnosis of CAP were mostly performed more than a decade ago. In recent years, modern PCR-based microbiological diagnostics have allowed more sensitive detection of viruses and atypical bacteria^6^. Therefore, the stratification of patients in our study is probably more accurate.

Several studies have shown that LUS is a useful tool for detecting CAP in children, especially when compared with CXR. A recently performed meta-analysis also confirmed the high sensitivity (96%) and specificity (93%) of LUS for detecting pneumonia in children^20^.

The potential of LUS to determine the aetiology of acute respiratory failure in adults admitted to the ICU has already been assessed previously, and the Bedside Lung Ultrasound in Emergency (BLUE) protocol has been established, which includes an algorithm according to which a consolidation with air bronchograms and/or focal accumulation of B-lines are associated with pneumonia^33^. This algorithm did not differentiate between the different microbial aetiologies of pneumonia and was later further upgraded for the purpose of differentiating between the viral LRTI and bacterial superinfection in children during the 2009 H1N1 influenza pandemic. In this study, a bacterial superinfection was considered in all patients in whom lung consolidation with an air bronchogram was detected using LUS. Small subpleural consolidations or confluent B-lines were considered as characteristics of viral pneumonia. Similarly, patients with an alveolar infiltrate on CXR were classified as having bacterial pneumonia, and viral pneumonia was assumed when interstitial infiltrates were detected on CXR^26^. In this way, a high correlation between LUS and CXR was determined. However, the potential of CXR for the microbial diagnosis of CAP is limited^2^, as more than half of patients with interstitial infiltrates on CXR were supposed to have bacterial CAP^13^. Therefore, we have not considered CXR as the “gold standard”, nor have we focused on the comparison between LUS and CXR. Our patients were stratified mainly according to the microbiological results. We considered bacterial co-infection or superinfection only in those patients with proven viral infection who had alveolar infiltrates on CXR and leucocytosis and an increased serum PCT value as described in the Methods section and shown in Fig. 1.

As an increased number of B-lines is also encountered in infants with bronchiolitis^34^, this finding alone was not considered proof of pneumonia in our study. Similarly, we did not include patients with small subpleural consolidations without hyperechoic and dynamic air bronchogram or without shred sign. In this way, we tried to exclude patients with viral bronchiolitis, but we probably also missed a few patients who, in similar studies^35^, would be diagnosed with viral pneumonia.

The median size of the (largest) LUS-detected consolidation in our study was 30 mm in bacterial CAP and 15 mm in viral CAP. The median size of the consolidation in the bacterial CAP was exactly the same as that presented by Urbankowska *et al*. In this study, a correlation between the size of the LUS-detected pneumonic consolidation and the neutrophil count was observed, although they did not distinguish between the different aetiologies of CAP^19^. We confirmed a correlation between WBC count and the size of the consolidation and observed a negative correlation between the WBC count and the number of consolidations, which both indicate the association of bacterial aetiology with large and solitary consolidations. Biagi *et al*. studied the usefulness of LUS for diagnosing bacterial superinfection in children (up to 2 years old) with acute bronchiolitis and found that bacterial superinfection is very likely when consolidations larger than 1 cm are detected^27^. According to our results, this threshold is relatively low, as we found that the median size of the (largest) consolidations in viral CAP was 15 mm. ROC curve analysis has shown that a cut-off of 21 mm consolidation size, as detected with LUS, yielded an optimal sensitivity of 80% and specificity of 75% for differentiating between the viral and bacterial CAP in the present study. We also included 12 (8.2%) patients who were treated with antibiotics before admission. This treatment could cause some regression of consolidations in the bacterial CAP group even before the first LUS was performed^24^. We assume that the differences in the size of the consolidations between the different types of CAP would be even more significant without those patients.

We detected multiple consolidations with LUS in 40.8% of all patients, which is comparable to the study performed by Caiulo *et al*., who found multiple consolidations in approximately 30% of patients^36^. In our study, multiple consolidations were detected in only 17.3% of patients with bacterial CAP and were significantly more common (65.4%) in patients with viral CAP. Although Caiulo *et al*. did not consider the microbial aetiology of CAP, the high percentage of multiple consolidations indicates that they included a substantial proportion of children with viral aetiology. The mean size of the consolidations measured in the study performed by Caiulo *et al*. was 18 mm, which also supports this presumption, as the value is very close to our results regarding the size of the consolidations in viral CAP. None of the abovementioned studies analysed the position of the consolidations (unilateral vs. bilateral). In our study, LUS-detected, bilateral consolidations were significantly more common in viral CAP (51.9%) than in bacterial CAP (8.0%). These results are in concordance with some of the previous studies using CXR, where bilateral infiltrates were associated with viral pneumonia^14^. The relatively low proportion of bilateral and/or multiple consolidation in bacterial CAP in our study may result from the exclusion of patients with severe cases of bacterial CAP, who are treated in the ICU. In such cases, multiple consolidations are expected to be more common^6^. Therefore, we suggest that LUS findings should always be interpreted in light of other clinical and laboratory findings. In our study, we detected bilateral consolidations with LUS in 28.6% of all patients, compared to 18.5% detected with CXR, which also indicates a higher sensitivity of LUS, although we observed a significant agreement between both imaging diagnostics regarding this issue.

Radiological findings in children with CAP may differ according to the age group^37^. As we included children across a wide age span, we analysed the influence of age on LUS characteristics (results not shown). We found no association except the positive correlation between the age and the size of the consolidations in the subgroup of patients with bacterial CAP. Therefore, we performed regression analysis adjusting for age and still found that the larger size of the consolidation was highly significant for the bacterial CAP.

Pleural effusion, which is considered highly specific for bacterial CAP, was detected with LUS in only 18.7% of our patients with bacterial CAP, which is a significantly higher proportion than that in viral CAP but not significantly different from that in atypical bacterial CAP. Previous studies reported that the prevalence of pleural effusion in adults admitted to the hospital because of CAP was 15–44%. These proportions are comparable to our findings, as bacteria are the most common cause of CAP in adults^38^.

We usually dismissed patients with CAP after 2–3 days of hospitalization, and the follow-up LUS was performed immediately before the dismissal. In our study, a regression of consolidations was observed in 96.6% of patients with bacterial CAP and in 33.3% of patients with viral aetiology. We detected no consolidations (complete resolution) at follow-up in 27.6% of patients with bacterial CAP and in only 2.6% of patients with viral CAP. In bacterial CAP, the median size of the consolidation diminished at follow-up LUS to 14.5 mm (from 30 mm) and in viral CAP to 12 mm (from 15 mm). Caiulo *et al*. followed patients for more than 14 days and observed a regression in 91.6% of patients^36^. Urbankowska *et al*. found a complete regression of pneumonic consolidations at follow-up between the 5^th^ and 7^th^ day in 44.0% of patients, and the median size of the (largest) consolidation diminished from 30 mm to 21 mm in that time interval^19^. The direct comparison of both studies with our results is difficult because we performed the follow-up LUS much earlier in the disease course. The observed proportion (and magnitude) of the regression of consolidations in both studies cited above is somewhere in between our results regarding the regression of bacterial and viral CAP and probably reflects the inclusion of patients with non-bacterial CAP^19,36^. A faster resolution of bacterial CAP in our study can be attributed to the effect of antibiotic treatment. Musolino *et al*. also performed follow-up LUS after 2 days of antibiotic treatment and found that pleural effusion disappeared in half of the cases^24^. LUS has proven to be useful tool for monitoring the effect of treatment on bacterial CAP. We observed a much faster regression of LUS-detected consolidations than previous studies that monitored the resolution of pneumonia with CXR. Radiographic resolution of pneumonia falls well behind the clinical cure assessed by physicians and was observed in only 30.8% of patients after 10 days^39^. Our results confirm that LUS is the preferred imaging method for the follow-up of CAP, as it almost parallels the clinical course. However, compared to the other analysed LUS characteristics (the size, number and position of consolidations), the findings regarding the course of pneumonic consolidations were of minor importance, as we aimed to improve the initial treatment decisions in patients with CAP.

To our knowledge, no other studies have examined the LUS characteristics of atypical bacterial pneumonia so far. According to our results, LUS findings in atypical bacterial pneumonia are similar to those in viral CAP. We observed that the consolidations in atypical bacterial CAP were more likely to be smaller, bilateral and multiple than those in bacterial CAP. With LUS, we detected multiple consolidations in 65.0% and bilateral consolidations in 45.0% of patients with atypical bacterial CAP. Our findings support the results of a study performed by Hsieh *et al*., where 50% of children with *M*. *pneumoniae* pneumonia presented with interstitial and bilateral infiltrates on CXR^15^. Considering the detection of bilateral infiltrates in atypical bacterial pneumonia, we found LUS to be superior to CXR for differentiating between atypical and bacterial CAP. Bilateral infiltrates were detected with CXR in only 25.0% of our patients with atypical bacterial CAP, which was not significantly more common than in classic bacterial CAP. We observed a slower regression of consolidations in patients with atypical bacterial CAP than in those with classic bacterial CAP. At follow-up, the regression of consolidations as determined by LUS was observed in only 50% of patients and complete resolution was observed in 14.3%, which is similar to that found in viral CAP and significantly less than we observed in classic bacterial CAP. In contrast to our results, a study performed by Bruns *et al*. with CXR in adults has shown the fastest resolution in atypical bacterial pneumonia caused by *M*. *pneumoniae*, followed by psittacosis and pneumococcal pneumonia^39^. However, a comparison of both imaging methods for this purpose is inappropriate because we performed follow-up examinations after a much shorter time period (two to three days) than the interval of a few weeks for CXR-based follow-up performed by Bruns *et al*.^39^. Regarding the differentiation of atypical from viral CAP with LUS, we found that the only difference was the larger size of the (largest) consolidation in patients with atypical bacterial CAP (median 20 mm). However, the patients with atypical bacterial CAP were significantly older (median 7 years) than those with viral CAP (median 2.2 years). According to our results, the combination of school age, the presence of crackles on auscultation, moderately increased WBC count and ultrasonic detection of multiple and/or bilateral consolidations of medium-size indicate an infection with atypical bacteria. The differentiation between the viral and atypical bacterial CAP is less important than the identification of classic bacterial CAP. Infection with *M*. *pneumoniae* is usually mild and self-limited in otherwise healthy children and can be treated similar to viral LRTI in outpatient settings^40^. As we included only 20 patients with atypical bacterial CAP, larger samples are warranted to ascertain the LUS characteristics of atypical bacterial CAP.

Our results confirm that the epidemiological, clinical and laboratory characteristics are useful in establishing the aetiology of CAP. However, signs that are more specific for bacterial CAP (diminished breath sounds, bronchial breathing) are seldom present and are therefore insensitive^6^. We confirmed that laboratory results are probably more accurate in the aetiological diagnosis of CAP, but unlike the clinical characteristics, they are useful only when the diagnosis of CAP is already established. LUS in the hands of an experienced clinician can simultaneously detect CAP and add useful information regarding the aetiological diagnosis. However, the usefulness of LUS is enhanced when used in combination with epidemiological, clinical and laboratory data.

We observed a relatively low sensitivity (77%) of CXR for the detection of pneumonia. However, our results are in accordance with the findings from previous studies that compared the sensitivity of LUS and CXR in children and adults with CAP^41–43^.

A large proportion of bacterial CAPs among our patients probably reflects a low (below 50%) vaccination rate against pneumococci even in preschool children in Slovenia and the inclusion of school age children in whom viral CAPs are less common^6,44^. Second, the relatively low proportion of viral CAPs is our conservative approach, as we did not include patients with small subpleural consolidations and/or B-lines detected with LUS as described above. This could also contribute to the similarities in the LUS features of viral and atypical bacterial CAPs in our study.

Our study has several limitations. There is no “gold standard” for the diagnosis of CAP in children, and the differentiation between viral bronchitis/bronchiolitis and viral pneumonia is somehow arbitrary^35^. Moreover, the microbial diagnosis of pneumonia in children is not easy to determine because we usually do not take specimens from the lower airways. The detection of respiratory viruses and atypical bacteria in the upper airways is not a direct proof of lower respiratory tract infection because prolonged viral shedding or asymptomatic colonization is common in children^5^. Furthermore, bacterial CAP often follows viral LRTI, and the viruses are still detectable at that time^45^. Methods to detect bacteria in CAP in children are even less sensitive or specific. Blood culture is positive in less than 10% of children with uncomplicated bacterial CAP^46,47^. Sputum is seldom obtained from preschool children, and the results are not specific^48^. In addition, even with the cooperation of another paediatric pulmonologist and the paediatric radiologist in the analysis of LUS cine loops, the problem of interobserver variability in LUS remains a potential confounder. By limiting the study to patients admitted to the hospital, we improved the diagnostic accuracy regarding the correct diagnosis of CAP and the determination of the aetiology. However, the exclusion of milder forms of CAP could represent a selection bias.

In conclusion, we have shown that LUS can add useful information regarding the aetiology of pneumonia in children and is especially useful in differentiating between viral and bacterial CAP, which is of the utmost importance for initial management. LUS itself is not enough for establishing the microbial diagnosis of CAP but can replace CXR for that purpose in most cases. LUS is especially useful when used in combination with epidemiological, clinical and laboratory data. LUS is harmless, easy to perform and widely available for bedside investigation. In comparison with the results of microbiological investigations, LUS results are immediately available to the clinician, who must decide about the initial empirical treatment. We predict that LUS will be established as the first-line imaging method in children with CAP and that it will replace CXR for that purpose in most patients. The inclusion of lung ultrasound in the guidelines for CAP management is already warranted. However, better standardization of the investigation is required in the future to diminish the large intra- and interobserver variability of the results.

## Supplementary information

Dataset 1

## Data Availability

All data have been added to this manuscript and the Supplementary Material section.
